# Analysis of Growing Season Normalized Difference Vegetation Index Variation and Its Influencing Factors on the Mongolian Plateau Based on Google Earth Engine

**DOI:** 10.3390/plants12132550

**Published:** 2023-07-04

**Authors:** Yujie Yan, Zhiming Xin, Xuying Bai, Hongbin Zhan, Jiaju Xi, Jin Xie, Yiben Cheng

**Affiliations:** 1 School of Soil and Water Conservation, Beijing Forestry University, Beijing 100083, China; yanyujie323@163.com (Y.Y.); xzmlkn@163.com (Z.X.); baibailucky324@163.com (X.B.); 2 The Sand Forestry Experimental Center, Chinese Academy of Forestry, Bayannur 015200, China; 3 Department of Geology and Geophysics, Texas A&M University, College Station, TX 77843, USA; zhan@geos.tamu.edu; 4 Department of Remote Sensing and Mapping, Space Star Technology Co., Ltd., Beijing 100086, China; xiej@cma.gov.cn; 5 National Meteorological Centre, China Meteorological Administration, Beijing 100081, China; xijj_cast503@sina.com

**Keywords:** Mongolian Plateau, desertification, vegetation coverage change, climate change, trend analysis

## Abstract

Frequent dust storms on the Mongolian Plateau have adversely affected the ecological environmental quality of East Asia. Studying the dynamic changes in vegetation coverage is one of the important means of evaluating ecological environmental quality in the region. In this study, we used Landsat remote sensing images from 2000 to 2019 on the Mongolian Plateau to extract yearly Normalized Difference Vegetation Index (NDVI) data during the growing season. We used partial correlation analysis and the Hurst index to analyze the spatiotemporal characteristics of the NDVI before and after the establishment of nature reserves and their influencing factors on the GEE cloud platform. The results showed that (1) the proportion of the region with an upwards trend of NDVI increased from 52.21% during 2000–2009 to 67.93% during 2010–2019, indicating a clear improvement in vegetation due to increased precipitation; (2) the increase in precipitation and positive human activities drove the increase in the NDVI in the study region from 2000 to 2019; and (3) the overall trend of the NDVI in the future is expected to be stable with a slight decrease, and restoration potential is greater for water bodies and grasslands. Therefore, it is imperative to strengthen positive human activities to safeguard vegetation. These findings furnish scientific evidence for environmental management and the development of ecological engineering initiatives on the Mongolian Plateau.

## 1. Introduction

The Mongolian Plateau, known for its complex geographical conditions and fragile ecological environment, is a highly sensitive and vulnerable region to global climate change [1]. It holds significant geo-strategic importance as the core area of the “Silk Road Economic Belt”, “Grassland Road”, and trans-Eurasian railway project in Northeast Asia [2]. With a relatively small human population, the Mongolian Plateau offers a nearly natural ecosystem, making it a unique area for studying vegetation phenology in response to climate change [3,4,5]. In recent years, the ecological environmental issues on the Mongolian Plateau, including grassland degradation and intensified drought, have gained widespread attention due to the intensification of global climate change activities [6,7].

During March and April 2023, China experienced 10 sand and dust weather events, with 9 of them classified as severe sandstorms and dust storms, all caused by the Mongolian cyclone [8]. Addressing the desertification of the Mongolian Plateau has become an urgent need. Understanding the changes in vegetation coverage across different land types and predicting future trends is crucial. Due to grassland degradation, the Mongolian Plateau has become a major source of sand and dust storms in Northern China [9].

The Normalized Difference Vegetation Index (NDVI) is an effective quantitative remote sensing monitoring index used to analyze large-scale vegetation coverage, phenological changes, and vegetation dynamics [10]. Surface vegetation coverage plays a significant role in reducing wind erosion, preventing the occurrence and large-scale development of sand and dust storms, and minimizing their impact [11,12]. By utilizing the ratio of band intensities, NDVI mitigates various sources of noise caused by cloud shadows, topographic and solar angle variations, and atmospheric conditions present in the visible red and infrared bands [13,14,15]. However, the open-loop structure of the NDVI equation, which lacks feedback mechanisms, still makes it susceptible to error and uncertainty under variable atmospheric and canopy background conditions [16,17]. Matsushita et al. found that the spatial variations in reflectance in single channels and the Enhanced Vegetation Index (EVI) were mainly due to topographic effects, while NDVI could eliminate or weaken these effects due to its band ratio format [18]. NDVI and the Soil-Adjusted Vegetation Index (SAVI) exhibit similar performance when considering the influence of soil background, but NDVI has a stronger ability to estimate sparse vegetation cover compared to SAVI [19]. Kumari et al. identified the potential interference of snow in NDVI analysis, but it does not explain the observed seasonal reversal of NDVI in many catchments [20]. Jiao et al. demonstrated a significant correlation between 23 vegetation indices and measured vegetation cover, with NDVI showing the highest correlation coefficient [21]. Bao et al. studied NDVI’s dynamics and its response to climate change, revealing a general reversal of NDVI trends on the Mongolian Plateau between 1982 and 2010 [22]. According to Sternberg et al. [23], extreme events during the 1999–2001 drought on the Mongolian Plateau were closely related to the decrease in vegetation NDVI dynamics. Batima et al. also observed a significant warming trend and a slight decrease in precipitation on the Mongolian Plateau [24]. However, Du’s research shows an increasing trend in precipitation during the growing season on the Mongolian Plateau and increased vegetation coverage, which may lead to some discrepancies with the aforementioned studies [25].

In this study, we used NDVI values derived from MODIS13A data spanning from 2000 to 2019 to analyze the spatiotemporal variations in vegetation NDVI during the growing season (April to October) on the Mongolian Plateau. Tang [26] found that the coordinate control point data from Advanced Very High Resolution Radiometer (AVHRR) sensors were sparse, whereas MODIS provided higher-density and more accurate latitude and longitude information. The lower resolution and longer revisit time of Himawari may hinder capturing small differences in vegetation cover. Although reflectance-based BRDF shape indicators may contain information about the anisotropic reflectance pattern of the land surface, they have limitations in identifying specific BRDF shapes [21]. Ghorbanian et al. [27] explored the impact of climate change on vegetation phenology in the Piemonte region in Italy using MODIS-NDVI data from 2001 to 2019. By employing the Sen-Mk significance test trend analysis method on the GEE platform, they could effectively reduce the influence of error values and abnormal data on the statistical results. Time-frequency analysis methods, such as wavelet and cross-wavelet analyses, have been widely used for monitoring climate and vegetation [28]. Least-squares wavelet analysis (LSWA) enables the detection of short- and long-duration signals with variable frequencies and amplitudes over time [29]. This method does not rely on data following a linear trend and is insensitive to missing data and abnormal values. Additionally, we utilized the Hurst index to predict future vegetation change trends. The goals were to comprehend the relationship between vegetation changes and climate change on the Mongolian Plateau in recent years and provide scientific evidence for long-term ecological protection. This study contributes to effectively monitoring precipitation stress and related vegetation dynamics, which is crucial for enhancing early warning systems and assessing risks associated with other natural disasters like droughts and sandstorms.

## 2. Results

### 2.1. Temporal and Spatial Variations in NDVI

#### 2.1.1. Interannual Variation Characteristics

**Figure 1:**
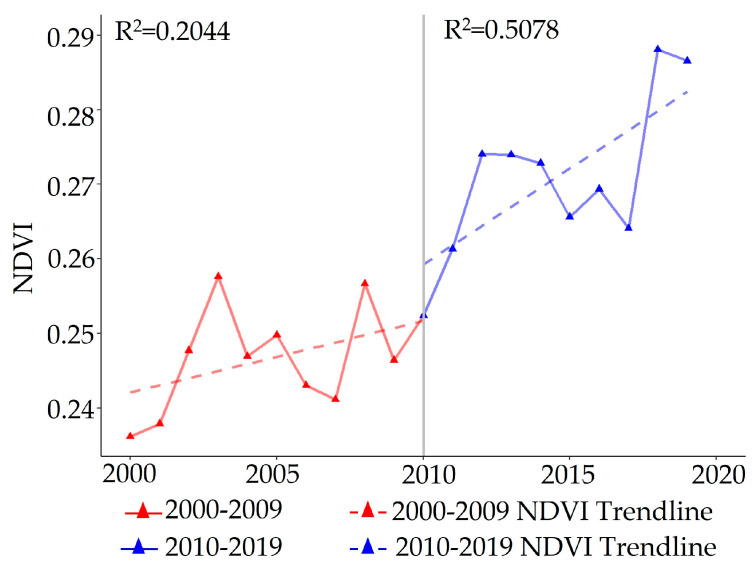
The NDVI trend during the growing seasons (2000–2019) on the Mongolian Plateau. The red line represents the NDVI trend from 2000 to 2009, while the blue line represents the NDVI trend from 2010 to 2019. These two trends exhibit noticeable differences in their growth rates.

In Figure 1, the average NDVI value during the growing season on the Mongolian Plateau shows slight fluctuations from 2000 to 2019, but shows a significant overall upward trend. The maximum NDVI value was recorded as 0.287 in 2019, while the minimum value was observed as 0.236 in 2000. The NDVI growth slope in the study region was 0.001 a^−1^ from 2000 to 2010, and this increased significantly to 0.0034 a^−1^ from 2010 to 2019, indicating clear acceleration in the growth rate and continuous vegetation recovery in Mongolia.

#### 2.1.2. Spatial Variation Characteristics

**Figure 2:**
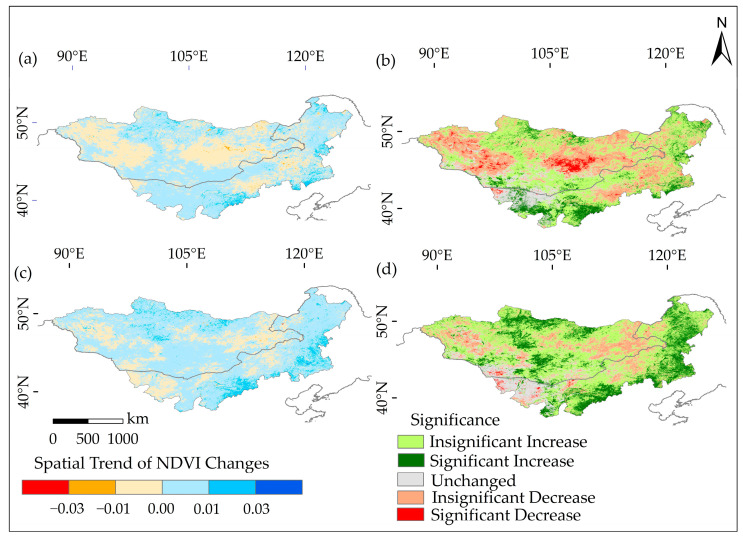
The spatial variations and significance tests of the growing season NDVI trends on the Mongolian Plateau. In particular: (**a**) shows the spatial variations in NDVI trends during the period from 2000 to 2009; (**b**) displays the significance testing of NDVI during the same period; (**c**) represents the spatial variations in NDVI trends from 2010 to 2019; (**d**) depicts the significance testing of NDVI during the same period. These figures provide visual representations of the changes in NDVI across the Mongolian Plateau, allowing for the analysis of spatial patterns and significance over the specified time periods.

**Figure 3:**
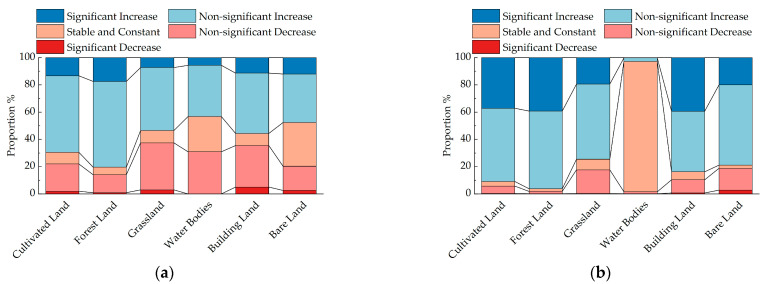
A figure illustrating the proportional representation of NDVI trends in different land types on the Mongolian Plateau. (**a**) shows a proportional representation of significance levels in the NDVI changes for different land types within the study region during the 2000–2009 period; (**b**) presents a proportional representation of significance levels in the NDVI changes for different land types within the study region during the 2010–2019 period.

From 2000 to 2009, approximately 31.01% of the Mongolian Plateau experienced a decrease in NDVI, with a significantly decreased region of 2.83%. These regions were predominantly located in the plateau’s desert regions (Figure 2). Conversely, around 52.21% of the total region witnessed an increase in NDVI, with a significantly increased region of 9.37%. This increase was primarily observed near the Kent Mountains in Mongolia, the Alxa League and Ordos grasslands in the Inner Mongolia Autonomous Region, and the Khorchin grasslands. The spatial pattern of NDVI changes during this period displayed a “decrease in the western and central regions, and increase in the eastern and southern regions”. Upon analyzing the land use types, the proportions of nonsignificant increases in cultivated land, forestland, grassland, water bodies, building land, and bare land from 2000 to 2009 were 56.11%, 62.96%, 46.22%, 37.53%, 44.30%, and 35.64%, respectively, indicating an overall NDVI increase across different land types. Between 2010 and 2019, the region with a decrease in NDVI accounted for 15.42% of the total region, with a significantly decreased region of only 0.65%. These regions were mainly concentrated in the southwestern part of the study region (Figure 2). Conversely, approximately 67.93% of the total region experienced an increase in NDVI, with a significantly increased region of 18.45%. These regions were predominantly distributed in the southeastern and northern regions of the Mongolian Plateau, suggesting an overall improvement in the vegetation’s NDVI. The spatial variation during this period exhibited a “decrease in the central region and increase in the southeastern region”. Analyzing land use types, the proportions of nonsignificant increases in cultivated land, forestland, grassland, water bodies, building land, and bare land from 2010 to 2019 were 53.70%, 56.99%, 55.14%, 2.59%, 44.40%, and 58.84%, respectively, indicating significant overall improvements in cultivated land, forestland, grassland, building land, and bare land (Table 1 and Figure 3).

### 2.2. Effects of Climate Factors on NDVI

#### 2.2.1. Interannual Variations in Climate Factors

**Figure 4:**
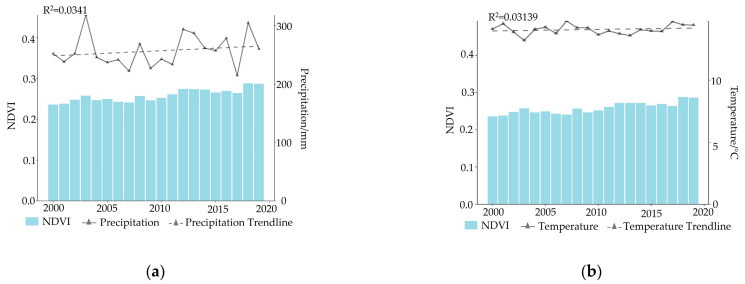
The characteristics of climate factor changes and NDVI variations from 2000 to 2019. (**a**) illustrates the patterns of precipitation changes and corresponding NDVI variations during this period, while (**b**) presents a graphical representation of temperature variations and their relationship with NDVI changes between 2000 and 2019.

The variation rate of precipitation on the Mongolian Plateau from 2000 to 2019 was 0.4 mm·a^−1^, indicating a slight upward trend overall but not reaching statistical significance (Figure 4). Conversely, the temperature remained relatively stable, with a growth rate of 0.016 °C·a^−1^ (Figure 4). Throughout this period, the climate on the plateau was generally arid, characterized by minor fluctuations in temperature but significant variations in precipitation. The consistent increase in precipitation has created favorable conditions for the growth and recovery of local vegetation, thus contributing to the observed increase in NDVI across the region.

#### 2.2.2. Relationship between Climate Factors and Vegetation NDVI: A Correlation Analysis

**Figure 5:**
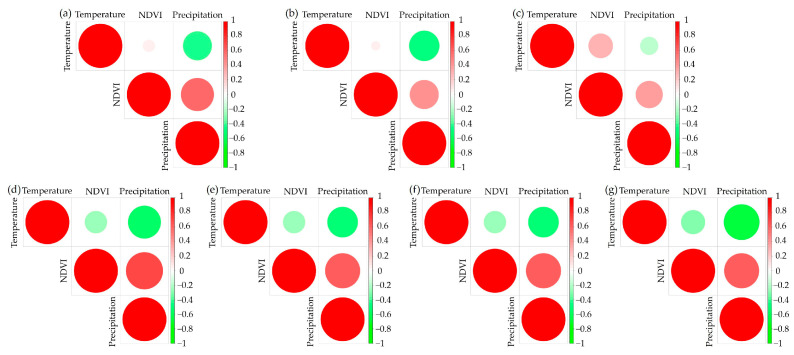
The correlations between NDVI, temperature, and precipitation between 7 ecoregions. (**a**) represents ecoregions 1: Temperate Broadleaf and Mixed Forests, (**b**) represents ecoregions 2: Temperate Conifer Forests, (**c**) represents ecoregions 3: Boreal Forests/Taiga, (**d**) represents ecoregions 4: Temperate Grasslands, Savannas, and Shrublands, (**e**) represents ecoregions 5: Flooded Grasslands and Savannas, (**f**) represents ecoregions 6: Montane Grasslands and Shrublands, (**g**) represents ecoregions 7: Deserts and Xeric Shrublands.

We conducted a correlation analysis between NDVI, precipitation, and temperature for the seven ecoregions on the Mongolian Plateau. The findings revealed a positive correlation between NDVI and precipitation. As shown in Figure 5, ecoregions 1–3 predominantly consist of forests, whereas ecoregions 4–7 primarily comprise grasslands, savannas, shrublands, and deserts. In ecoregions 1–3, there is no noticeable correlation between temperature and NDVI. However, a weak negative correlation can be observed between temperature and NDVI in ecoregions 4–7. Moreover, there exists a positive correlation between precipitation and NDVI in ecoregions 1–7, which is particularly prominent in ecoregions 4–7. The variation across different ecoregions can be attributed to the discrepancy in vegetation growth rates between forests and grasslands, savannas, shrublands, and deserts. The latter exhibit higher vegetation growth rates, leading to more sensitive responses from ecosystems. Additionally, grasslands, savannas, shrublands, and deserts are subjected to greater exposure to solar radiation and temperature fluctuations. It is worth noting that forests and grasslands, savannas, shrublands, and deserts display relative sensitivity to precipitation. These results are consistent with the overall patterns observed across the plateau.

**Figure 6:**
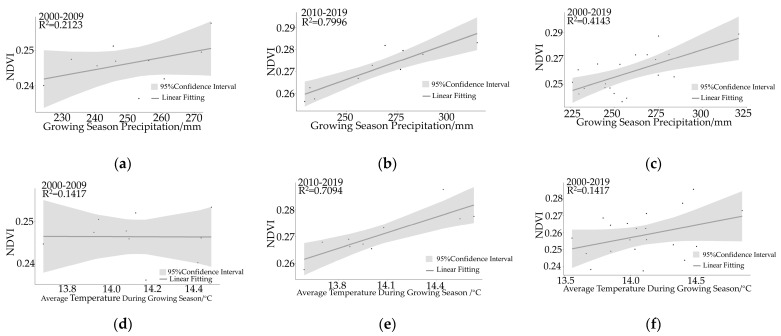
The correlation between NDVI and precipitation or temperature. (**a**) describes the relationship between the precipitation and NDVI during 2000–2009, (**b**) shows the relationship between the precipitation and NDVI during 2010–2019, (**c**) depicts the relationship between the temperature and NDVI during 2000–2009, (**d**) presents the relationship between the temperature and NDVI during 2000–2009, (**e**) illustrates the relationship between the temperature and NDVI during 2010–2019, and (**f**) represents the relationship between the temperature and NDVI during 2000–2019.

**Figure 7:**
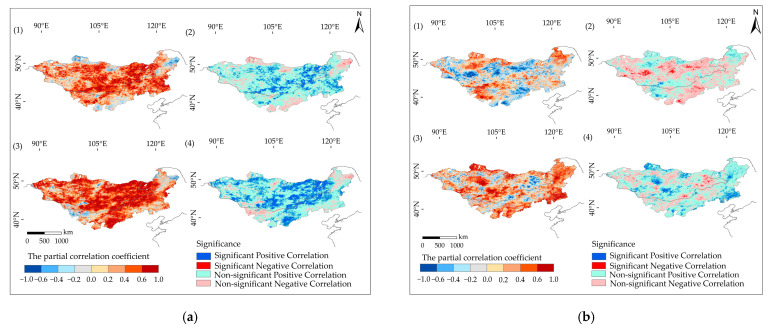
The partial correlation coefficients and significance test of the relationship between NDVI and precipitation or temperature during the growing season. (**a**) describes the relationship between NDVI and precipitation, (**b**) describes the relationship between NDVI and temperature. (**1**) represents the relationship during 2000–2009, (**2**) represents the partial correlation coefficients during 2000–2009, (**3**) represents the relationship during 2010–2019, and (**4**) represents the partial correlation coefficients during 2010–2019.

Figure 6 illustrates the correlation between precipitation and NDVI from 2000 to 2019, displaying the spatial variations in this correlation during the two time periods. During the period of 2000 to 2009, there was a mostly positive correlation between NDVI and precipitation. A nonsignificant positive correlation was observed in 65.89% of the study region, while regions with a significant positive correlation accounted for 15.77% of the total study region, primarily concentrated in the central and eastern regions, with fewer sites in the western region (Figure 7). The correlation coefficient between precipitation and NDVI during 2000–2009 was 0.21, indicating a significant positive correlation between precipitation and NDVI (*p* < 0.05). When precipitation was low, the NDVI tended to decrease, suggesting that annual precipitation had an impact on vegetation cover to some extent.

From 2010 to 2019, a nonsignificant positive correlation between NDVI and precipitation accounted for 59.32% of the total region. The regions with a significant positive correlation accounted for 17.23% and were primarily concentrated in the central, eastern, and southern regions, with some variations observed in the western region (Figure 7). This indicates that the influence of precipitation on NDVI has expanded in both degree and spatial extent. Furthermore, the correlation coefficient increased to 0.7966, surpassing that of the 2000–2009 period, and the NDVI exhibited significant increases during this time period. Through investigations, it was discovered that mining activities and afforestation on the Mongolian Plateau could introduce long-term errors. To mitigate these errors, partial correlation analysis was conducted on the precipitation and NDVI data for different time periods. The results demonstrated that precipitation was the primary factor driving vegetation growth and recovery.

As shown in Figure 6 and Figure 7, the average temperature during the growing season from 2000 to 2019 was recorded as 14.09 °C. The analysis of the partial correlation between temperature and NDVI revealed that temperature does not significantly impact NDVI (*p* < 0.05), indicating that it is not the primary driving factor for NDVI variations over a long-term time series in the study region (Figure 7). The correlation between temperature and NDVI differed between the two time periods.

During the period of 2000–2009, the average temperature during the growing season was 14.12 °C. Nonsignificant negative correlations accounted for 55.16%, nonsignificant positive correlations accounted for 46.36%, and significant positive correlations were observed in only 2.4% of the study region. The partial correlation analysis revealed that temperature was not the causal factor behind the changes in vegetation NDVI during this period.

From 2010 to 2019, the average temperature during the growing season was 14.07 °C. Nonsignificant negative correlations accounted for 31.29%, nonsignificant positive correlations accounted for 65.05%, and significant positive correlations were observed in 10.16% of the study region. The regions with significant positive correlations were primarily concentrated in the Khorchin grassland of Inner Mongolia and near the Hangai Mountains in Mongolia. The partial correlation analysis indicated a correlation coefficient of 0.7049 between temperature and NDVI, which was significantly higher than that observed from 2000 to 2009. Despite a slight decrease in average temperature, it still influenced vegetation recovery and growth during the period of 2010–2019, suggesting that a certain degree of temperature reduction could promote vegetation growth and recovery.

### 2.3. Future Trend of NDVI Variation

**Figure 8:**
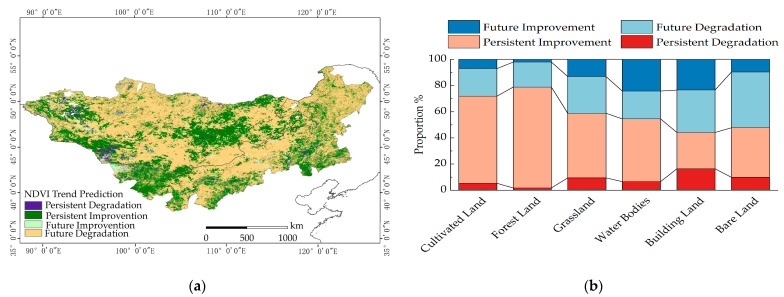
The spatial distribution of future NDVI trend and its proportions in different land types. (**a**) presents a spatial distribution map of the future NDVI trend; (**b**) displays the proportions of the future NDVI trend in different land types.

Using NDVI grid data, the Hurst exponent was calculated for each grid to predict the future trend of the NDVI. The average Hurst exponent for the entire region was found to be 0.47, denoted as H < 0.5, indicating weak anti-persistence in the NDVI over the entire Mongolian Plateau in the future. This suggests that the NDVI will exhibit small fluctuations and a slightly degraded trend in the future. Based on the analysis of the NDVI variation trend and the Hurst exponent data, the future NDVI trend was categorized into four distinct groups: persistent degradation, persistent improvement, future degradation, and future improvement. The proportion of regions classified as having persistent degradation was 2.10%, while those with persistent improvement accounted for 34.08% of the study region. Areas of future degradation were estimated to cover 61.16% of the region, while those of future improvement encompassed 2.66% (Figure 8).

According to Table 2, the highest proportion of future NDVI degradation on the Mongolian Plateau is observed in the bare land category, accounting for 37.94%. This is followed by the degradation proportion in buildings, which is ranked second at 32.41%. In terms of continuous degradation, the highest proportion is found in bare land at 16.46%, followed by barren land at 9.83%. Investigations have revealed the presence of severe cases of industrial and mining development, as well as desertification, on the Mongolian Plateau. Therefore, it is crucial to implement responsible industrial and mining practices and take measures to protect bare land through artificial vegetation and afforestation. These actions can help mitigate the degradation of plant NDVI and promote the recovery of vegetation in the region.

In terms of the future improvement of NDVI, water bodies have the highest proportion at 24.29%, followed by buildings at 23.43%. Regarding continuous improvement, cultivated land has the highest proportion at 66.50%, followed by forestland at 77.07%. These findings indicate that measures such as artificial planting, cultivation, and the protection of water bodies have been effective in increasing the local vegetation NDVI on the Mongolian Plateau, promoting vegetation growth and restoration.

The vegetation growth status on the Mongolian Plateau is not stable in the long term, as the future trend of NDVI changes differs from the past trend. Human activities on the plateau, such as overgrazing, deforestation, and land-use changes, have had detrimental effects on grassland ecosystems, resulting in vegetation degradation, land desertification, and land erosion. These changes may also be influenced by the inconsistent effectiveness of water and soil conservation measures and grassland protection policies, or the impact of random interference factors. It is crucial to address these issues and implement sustainable practices to ensure the long-term health and restoration of vegetation on the Mongolian Plateau.

## 3. Discussion

Desertification has become a significant issue on the Mongolian Plateau, influenced by both climate change and human activities [30]. Climate change affects the vegetation environment, thereby impacting vegetation growth [31]. However, according to Tong’s research [32], the region exhibited decreasing and increasing drought trends in 72.2% and 27.8% of the regions, respectively. This indicates an overall humidification trend on the Mongolian Plateau, which would have a positive impact on the NDVI growth of vegetation. Nevertheless, research in this region has focused less on the NDVI of specific land cover types. The vegetation phenology characteristics of Mongolia and their responses to global changes are sensitive and complex [3,33]. Further analysis is required to investigate the phenological characteristics of different vegetation types and their differential responses to climate and topography. Additionally, arid climate zoning research in Mongolia is needed [34,35]. Predictions by Amy E. Hessl et al. on future drought trajectories in Mongolia have considerable uncertainty, depending on factors such as increased evapotranspiration demand, precipitation expectations, and the physiological responses of vegetation to rising CO_2_ [36]. In this study, the change in vegetation cover NDVI was quantitatively analyzed to understand the relationship between the degree of drought change and climate change, with a focus on the Mongolian Plateau.

Using the GEE platform, this study monitored the long-term dynamic changes in vegetation on the Mongolian Plateau using Landsat remote sensing imagery from 2000 to 2019. The results showed that regions with an increasing NDVI accounted for 67.93% of the total study region, indicating significant improvement in the vegetation’s ecological environment. This finding aligns with the research results of Luo et al. [37]. Leveraging Landsat remote sensing imagery and the GEE integrated operational environment allowed for vegetation monitoring results with a long time span and high spatial resolution [38]. This approach accurately characterizes the spatiotemporal evolution of vegetation NDVI and enhances the technical means for long-term and small-area vegetation monitoring. The study results indicate that the warming and drying climate explains the decrease in NDVI in the study region, which is consistent with the research results of Chen Shujun et al. [39]. Increased temperature and reduced precipitation can lead to meteorological drought, causing water shortages in the atmosphere and soil, exacerbating vegetation drought stress, and inhibiting vegetation growth [40]. Previous studies have shown that climate warming is the main factor influencing vegetation NDVI changes in many areas of the middle and high latitudes of the Northern Hemisphere [41,42,43,44,45]. Bao et al. studied the NDVI and found a positive correlation with precipitation but a weak correlation with temperature, implying that precipitation amounts in the growing season are a key factor regulating vegetation dynamics at the plateau scale. This conclusion aligns with the current analysis [22]. In this study, NDVI changes during the vegetation growing season in Mongolia were primarily influenced by precipitation changes. This result may be related to Mongolia’s location in semiarid and arid regions [46,47,48]. The study also predicted future NDVI changes in the study region, indicating an overall relatively stable trend with a slight possibility of decline, which may be attributed to overgrazing and human activities such as industrial and mining development [25].

However, this study only focuses on the influence of two climate factors, temperature and precipitation, on NDVI. It does not consider other potential climate factors such as evapotranspiration. Therefore, future research needs to comprehensively consider the combined effects of multiple climate factors on vegetation NDVI and utilize multiple sources of land cover data to construct more accurate samples when studying NDVI trend changes in different land cover types. This study also does not fully account for the lag in correlation and the limitations of satellite data sources. In order to better understand the trends in vegetation NDVI on the Mongolian Plateau, future studies should delve into the driving mechanisms of human activities on vegetation NDVI changes by closely integrating the specific timing and direction of ecological engineering implementation in the study region.

## 4. Materials and Methods

### 4.1. Study Region

**Figure 9:**
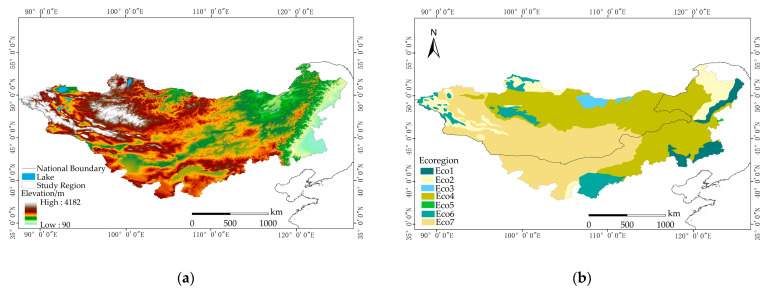
(**a**) The location of the study region and its corresponding elevation map. (**b**) The ecoregions of the Mongolian Plateau; the Mongolian Plateau comprises various ecoregions with distinct ecological characteristics. The ecoregions identified on the Mongolian Plateau are as follows: Eco1: Temperate Broadleaf and Mixed Forests, Eco2: Temperate Conifer Forests, Eco3: Boreal Forests/Taiga, Eco4: Temperate Grasslands, Savannas, and Shrublands, Eco5: Flooded Grasslands and Savannas, Eco6: Montane Grasslands and Shrublands, Eco7: Deserts and Xeric Shrublands. These ecoregions represent different vegetation types and ecological communities found on the Mongolian Plateau.

We chose the Mongolian Plateau as our study region, which is situated in Northeastern Asia, and stretches from the Greater Khingan Range in the east to the Altai Mountains in the west. It is bordered by the Sayan Mountains, the Kent Mountains, and the Yablonoi Mountains to the north. On the southern side, it extends along the Great Wall and the Yin Mountains [49,50]. This region encompasses the entire territories of Mongolia, Southern Russia, and Northern China. The study region included the main portion of the Mongolian Plateau in Mongolia and the Inner Mongolia autonomous region of China (Figure 9). Geographically, it spans between 37°22′–53°20′ N and 87°43′–126°04′ E. The region is characterized by significant variations in relief, with land slopes gradually decreasing from west to east. The topography and landforms are diverse, consisting of mountains, hills, plateaus, and plains [4]. Vegetation distribution follows a belt-like pattern, with the Gobi Desert, grasslands, and forests extending from west to east [51].

### 4.2. Datasets and Pre-Processing

The NDVI data used in this study were obtained from the MOD13A1 product of MODIS (Moderate Resolution Imaging Spectroradiometer), which provides 16-day reflectivity data with a spatial resolution of 500 m. The data were obtained from the data center of the National Aeronautics and Space Administration (NASA) in the United States. You can access the data at the following link: https://www.nasa.gov/ (accessed on 1 April 2023). The digital map of European ecological regions (DMEER) used in this study was provided by the European Environment Agency. You can find the map at the following link: https://www.eea.europa.eu/data-and-maps/data/digital-map-of-european-ecological-regions (accessed on 17 June 2023). For climate data, the annual average temperature and annual precipitation data were obtained from the ERA5 dataset, which is the fifth generation of the European Centre for Medium-Range Weather Forecasts (ECMWF) global atmospheric climate analysis dataset. The dataset can be accessed at: https://cds.climate.copernicus.eu/ (accessed on 1 April 2023). The resolution of the climate data was 0.25° × 0.25°.

The remote sensing data were primarily processed using the Google Earth Engine (GEE) cloud platform, which is suitable for processing long-term time series data. Landsat 5, 7, and 8 satellite datasets were selected for calculating vegetation coverage. The datasets were imported into the GEE platform and corrected for platform synthesis. They were then filtered for cloud cover and shadows. Vegetation coverage was derived by fusing the red band and near-infrared band and calculating the NDVI.

The study region vector data were imported, projected, and stitched together to select the study period and output pixel values for calculating vegetation coverage. Monthly NDVI data were synthesized using the maximum composite method. The NDVI values during the growing season, which spans from April to October, were calculated. Land type data were created by cropping the GlobeLand3 global land cover data. The DEM (Digital Elevation Model) data used in this study were obtained from NASADEM_HGT, with a spatial resolution of 30 m.

### 4.3. Methods

#### 4.3.1. Sen’s Slope Estimator Method for Trend Analysis (Sen-Mk Trend Analysis)

Sen-Mk trend analysis for trend detection is composed of two methods: the Theil–Sen median method for calculating the change trend of data in a time series and the Mann–Kendall nonparametric statistical test method for assessing the significance of the trend. The computation formulas are as follows:

(1) $$S=median\left(\frac{x_{j}-x_{i}}{j-i}\right)\forall j>i$$

The trend degree *S* is used to determine the rising or falling trend of a time series. When *S* > 0, the NDVI shows an upwards trend over time, and vice versa for *S* < 0.

The MK test for trend significance is also a nonparametric statistical test that does not rely on the assumption of normal distribution or a linear change trend in the data. The computation formulas are as follows:

(2) $$Z=\left\{\begin{array}{c} \frac{S-1}{\sqrt{Var\left(S\right)}}\left(S>0\right)\\ 0\left(S=0\right)\\ \frac{S+1}{\sqrt{Var\left(S\right)}}\left(S<0\right) \end{array}\right.$$

(3) $$S=\sum_{i=1}^{n-1}\sum_{j=i+1}^{n}sgn(x_{j}-x_{i})$$

(4) $$sgn\left(\theta \right)=\left\{\begin{array}{c} 1,\;\theta >0\\ 0,\;\theta =0\\ -1,\;\theta <0 \end{array}\right.$$

(5) $$Var\left(S\right)=\frac{n(n-1)(2n+5)}{18}$$

where *n* represents the length of the time series and *sgn* is the sign function. The *Z* statistic ranges from negative infinity to positive infinity. The null hypothesis is that there is no trend in the sequence when a two-sided trend test carried out. Given a significance level α, if |*Z*| > *Z*_α_, then the null hypothesis is rejected, indicating a significant trend in the sequence. Conversely, there is no significant trend.

According to the NDVI trend classification of Yuan et al. [52], when α = 0.05, that is, |*Z*| ≥ 1.96, the NDVI time series change trend is significant. In this paper, according to the trend level standard, it was categorized into 5 levels: significant increase (*S* ≥ 0.0005, |*Z*| ≥ 1.96), nonsignificant increase (*S* ≥ 0.0005, |*Z*| < 1.96), basically stable (*S* < 0.0005, |*Z*| < 1.96), nonsignificant decrease (*S* < −0.0005, |*Z*| < 1.96), and significant decrease (*S* < −0.0005, |*Z*| ≥ 1.96).

#### 4.3.2. Partial Correlation Analysis

Partial correlation analysis refers to analyzing the correlation between two variables while controlling for the influence of a third variable. The strength of the net correlation between the two variables is determined by the partial correlation coefficient [53]. The calculation formula is as follows:

(6) $$r_{xy,\;z}=\frac{r_{xy}-r_{xz}r_{yz}}{\sqrt{1-r_{xz}^{2}}\sqrt{1-r_{yz}^{2}}}$$

where *x*, *y*, and *z* are three variables. The *r_xy_*, *r_xz_*, and *r_yz_* coefficients are the partial correlation coefficients between variable *x* and variable *y*, variable *x* and variable *z*, and variable *y* and variable *z*, respectively. Based on the calculated *p* values and correlation coefficients, the correlation was classified into one of four categories: significantly positive (*r* > 0, *p* < 0.05), not significantly positive (*r* > 0, *p* > 0.05), significantly negative (*r* < 0, *p* < 0.05), or not significantly negative (*r* < 0, *p* > 0.05).

#### 4.3.3. Hurst Exponent Analysis

The Hurst exponent was used to analyze and study the future trend in the NDVI changes in a given region. There are several methods to calculate the Hurst exponent, and this paper adopted the commonly used R/S analysis method. The calculation formula is as follows:

Given a time series of *NDVI*_(*t*)_ where *t* = 1, 2, …, n, and any positive integer *T* ≥ 1, the summation range is 1 ≤ *t* ≤ *T*, and the mean sequence is defined as:

(7) $${\overset{-}{NDVI}}_{\left(T\right)}=\frac{1}{T}\sum_{t=1}^{T}NDVI_{\left(t\right)}$$

The cumulative deviation is:

(8) $$X_{\left(t,T\right)}=\sum_{i=1}^{t}(NDVI_{\left(i\right)}-{\overset{-}{NDVI}}_{\left(T\right)})\;1\leq t\leq T$$

The range is:

(9) $$R_{\left(T\right)}=\underset{1\ll t\ll T}{\max}X_{\left(t,T\right)}-\underset{1\ll t\ll T}{\min}X_{\left(t,T\right)}$$

The standard deviation is:

(10) $$S_{\left(T\right)}={\left[\frac{1}{T}\sum_{t=1}^{T}{\left(NDVI_{\left(t\right)}-{\overset{-}{NDVI}}_{\left(T\right)}\right)}^{2}\right]}^{\frac{1}{2}}\;1\leq t\leq T$$

According to the above formula, *R*_(*T*)_/*S*_(*T*)_ ≅ *R*/*S*, and the Hurst exponent H is obtained by fitting the equation log(*R*/*S*)_n_ = a + H × log(n). If 0.5 < H < 1, it indicates that the NDVI time series exhibits positive persistence, and the stronger the persistence, the closer H is to 1; If H = 0.5, it means that the NDVI trend in the time series is a random sequence without persistent changes; If 0 < H < 0.5, it indicates that the NDVI trend in the time series exhibits negative persistence, meaning that the future trend is opposite to the past trend, and the stronger the negative persistence, the closer H is to 0.

## 5. Conclusions

In 2023, the Mongolian Plateau experienced 13 instances of dust storms. We aim to uncover the environmental changes taking place on the Mongolian Plateau. This study provides an in-depth analysis of the changes in NDVI during the growing season and their influencing factors in the central part of the Mongolian Plateau. Additionally, it offers basic predictions for future NDVI trends and their distribution across different land types. The main conclusions drawn from this study are as follows:

1. Between 2000 and 2009, the vegetation NDVI showed a gradual increase at a rate of 0.001 per year, resulting in an overall increase of 52.21% in the study region. From 2010 to 2019, the vegetation NDVI exhibited a faster growth rate of 0.0034 per year, leading to a larger increase of 67.93% of the total study region. These findings indicate the successful recovery of vegetation on the Mongolian Plateau.
2. An analysis of climate trends from 2000 to 2009 revealed a slight humidification trend in the study region. The NDVI showed no significant correlation with temperature but exhibited a positive and significant correlation with precipitation, accounting for 15.77% of the region. Barren land experienced the most significant increase at 12.03%. Forestland showed the highest increase at 62.96%, although it was not statistically significant. Furthermore, the proportion of construction land significantly decreased by 5.06%, potentially due to excessive industrial and mining development. Between 2000 and 2010, the NDVI demonstrated positive correlations with both precipitation and temperature, with significant positive correlations of 17.23% and 10.16%, respectively. This suggests that during this period, precipitation played a more significant role in NDVI growth, while the effect of temperature was weaker. Forestland showed the most significant increase at 19.37%, while barren land exhibited the highest increase both significantly (2.77%) and insignificantly (58.84%). These results indicate that measures such as grass planting and other artificial methods to promote vegetation coverage may have contributed to the increase in NDVI.
3. The NDVI trend from 2000 to 2019 suggests that there will be opposite and sustained development compared to the current growth trend, with an expected degradation rate of 61.16%. Therefore, ecological protection and vegetation restoration are of utmost importance. Implementing artificial measures such as grass planting is necessary to increase vegetation NDVI, while also prioritizing the protection of grassland, forestland, and water bodies, and addressing degradation in barren land and construction land. To prevent future degradation, a series of measures, including controlling overgrazing, strengthening grassland protection, improving land management, and implementing appropriate agricultural and pastoral practices, will be necessary. Additionally, enhancing scientific research and innovation, promoting a virtuous circle of natural ecology and human economic and social development, and protecting the ecological environment of the Mongolian Plateau are crucial.

## Figures and Tables

**Table 1:** The statistical analysis of NDVI change trends in different land types during the 2000–2009 and 2010–2019 periods on the Mongolian Plateau includes the calculation of the area and proportion represented by each trend.

Year	Trend of Change	Nonsignificant Decrease		Significant Decrease		Stable and Constant		Nonsignificant Increase		Significant Increase	
		Area/km^2^	Proportion/%	Area/km^2^	Proportion/%	Area/km^2^	Proportion/%	Area/km^2^	Proportion/%	Area/km^2^	Proportion/%
2000 – 2009	Cultivated Land	12,868.70	20.07	1247.97	1.95	5456.50	8.51	35,978.26	56.11	8573.59	13.37
	Forest Land	9021.93	13.11	666.49	0.97	3785.99	5.50	43,329.14	62.96	12,014.57	17.46
	Grassland	601,570.23	34.53	51,820.90	2.97	156,326.06	8.97	805,276.55	46.22	127,378.97	7.31
	Water Bodies	5719.60	30.87	1.85	0.01	4803.16	25.92	6953.28	37.53	1050.51	5.67
	Building Land	1236.42	30.49	205.24	5.06	356.53	8.79	1796.43	44.30	460.93	11.37
	Bare Land	151,620.02	17.80	21,433.82	2.52	272,774.72	32.02	303,599.03	35.64	102,452.02	12.03
2010 – 2019	Cultivated Land	3156.74	5.40	206.24	0.35	2002.30	3.43	31,385.23	53.70	21,696.44	37.12
	Forest Land	1219.78	1.77	70.39	0.10	1339.91	1.95	39,238.18	56.99	26,980.53	39.19
	Grassland	319,802.26	17.49	3108.89	0.17	143,332.76	7.84	1,008,258.24	55.14	354,163.07	19.37
	Water Bodies	4373.65	1.46	168.81	0.06	286,030.66	95.66	7750.88	2.59	672.06	0.22
	Building Land	397.60	9.71	29.09	0.71	237.81	5.81	1818.14	44.40	1612.16	39.37
	Bare Land	78,497.02	16.01	13,561.24	2.77	11,793.24	2.40	288,556.33	58.84	98,040.56	19.99

**Table 2:** A table showing the proportions of the future NDVI trend in different land types.

Trend of Change	Future Improvement	Future Degradation	Persistent Improvement	Persistent Degradation
	Proportion/%	Proportion/%	Proportion/%	Proportion/%
Cultivated Land	5.35	66.50	7.10	21.06
Forest Land	1.74	77.07	2.09	19.10
Grassland	9.40	49.45	13.15	27.99
Water Bodies	6.59	47.97	24.19	21.25
Building Land	16.46	27.70	23.43	32.41
Bare Land	9.83	37.94	9.67	42.57

## Data Availability

All the data are available from the corresponding author on reasonable request.
